# Individuality in harpsichord performance: disentangling performer- and piece-specific influences on interpretive choices

**DOI:** 10.3389/fpsyg.2013.00895

**Published:** 2013-11-28

**Authors:** Bruno Gingras, Pierre-Yves Asselin, Stephen McAdams

**Affiliations:** ^1^Department of Cognitive Biology, University of ViennaVienna, Austria; ^2^Schulich School of Music, McGill UniversityMontreal, QC, Canada

**Keywords:** music performance, individuality, expressive strategies, harpsichord, interpretation, concordance

## Abstract

Although a growing body of research has examined issues related to individuality in music performance, few studies have attempted to quantify markers of individuality that transcend pieces and musical styles. This study aims to identify such meta-markers by discriminating between influences linked to specific pieces or interpretive goals and performer-specific playing styles, using two complementary statistical approaches: linear mixed models (LMMs) to estimate fixed (piece and interpretation) and random (performer) effects, and similarity analyses to compare expressive profiles on a note-by-note basis across pieces and expressive parameters. Twelve professional harpsichordists recorded three pieces representative of the Baroque harpsichord repertoire, including three interpretations of one of these pieces, each emphasizing a different melodic line, on an instrument equipped with a MIDI console. Four expressive parameters were analyzed: articulation, note onset asynchrony, timing, and velocity. LMMs showed that piece-specific influences were much larger for articulation than for other parameters, for which performer-specific effects were predominant, and that piece-specific influences were generally larger than effects associated with interpretive goals. Some performers consistently deviated from the mean values for articulation and velocity across pieces and interpretations, suggesting that global measures of expressivity may in some cases constitute valid markers of artistic individuality. Similarity analyses detected significant associations among the magnitudes of the correlations between the expressive profiles of different performers. These associations were found both when comparing across parameters and within the same piece or interpretation, or on the same parameter and across pieces or interpretations. These findings suggest the existence of expressive meta-strategies that can manifest themselves across pieces, interpretive goals, or expressive devices.

## Introduction

Over the last few decades, a growing body of research has examined issues related to individuality in musical performance (e.g., Repp, 1992; see Sloboda, 2000 for a review). Computational methods have led to the development of higher-level descriptors to capture and identify recurrent expressive gestures associated with a given performer (Widmer and Goebl, 2004; Saunders et al., 2008). However, few studies have attempted to quantify markers of individuality that transcend specific pieces and musical styles. Indeed, it seems likely that, among the factors which influence a performer's interpretive choices, some derive from performer-specific tendencies, including kinematic and neuromuscular “fingerprints” (Dalla Bella and Palmer, 2011; Van Vugt et al., 2013), whereas others stem from stylistic considerations related to the piece (or genre) being performed. In order to identify which performance characteristics are reliable markers of a performer's artistic individuality across genres and styles, it is necessary, as a first step, to disentangle these two contributions. Nevertheless, it has proven difficult, for several reasons, to untangle these factors. One obvious issue is that pieces vary in length, texture, and meter. Another issue is that these markers of artistic individuality may plausibly encompass several expressive parameters, such as articulation, velocity, or timing, instead of being restricted to a single expressive device. To identify such expressive “meta-strategies,” it is necessary to adopt a statistical approach suitable for analyzing parameters that are measured in different units. Thus, there is a need for a robust methodological approach that allows us to obtain valid statistical inferences even when comparing individual performance profiles across pieces and expressive parameters.

Stamatatos and Widmer (2005) showed, by developing a machine-learning approach based on a set of classifiers that could reliably differentiate among 22 pianists playing two pieces composed by Chopin, that performer-specific characteristics that are not tied to a particular piece could be identified from a symbolic representation (MIDI data) of the expressive parameters associated with each note. More recently, similar methods were successfully applied to the recognition of performers in commercial jazz recordings (Ramirez et al., 2010) and violin recordings (Ramirez et al., 2011) on the basis of the audio signal. In contrast to these studies, which focused mostly on the development of efficient algorithms for the automatic recognition of performers, the present article aims to expand this field of research in a different direction, by developing reliable and statistically rigorous methods for discriminating between piece-specific and performer-specific stylistic influences and for detecting commonalities in expressive patterns across pieces and interpretations.

Although a substantial body of empirical research has focused on piano performance (see Gabrielsson, 2003 for a review), there is a dearth of quantitative studies on expressive strategies in harpsichord performance. However, the study of harpsichord performance is particularly relevant in that it affords an opportunity to compare and extend the findings from piano performance research to other keyboard instruments that may favor different expressive strategies, as well as to musical genres that have been comparatively neglected in performance research. Here, we analyzed a set of recordings of three pieces played by twelve professional harpsichordists on an authentic Italian-style harpsichord equipped with a MIDI console which allowed the precise measurement of performance parameters. The three pieces selected for this study were representative of the Baroque harpsichord repertoire and covered a broad stylistic range: the third variation from the *Partita No. 12 sopra l'aria di Ruggiero* by Girolamo Frescobaldi (1583–1643), the *Prélude non mesuré No. 7*, an unmeasured prelude by Louis Couperin (1626–1661), and *Les Bergeries*, a rondo by François Couperin (1668–1733). The variation from the *Partita No. 12* (hereafter *Partita*) exemplifies the polyphonic, contrapuntal writing of the early Baroque period. The *Prélude non mesuré* (hereafter *Prélude*) belongs to a semi-improvised French harpsichord genre in which the notated score specifies the ordering and pitch height of the notes, but does not indicate measures, nor individual note durations in most cases (including the *Prélude*), thus giving performers more freedom to form their own interpretation and making this a particularly appropriate genre for research on individuality in performance. Finally, the *Bergeries* is typical of the early eighteenth century French harpsichord school, with François Couperin being probably one of its greatest exponents.

Besides examining recordings of three different pieces, we also compared different interpretations of the same piece by the same set of performers. Indeed, performers were invited to record three different interpretations of the *Partita*, each emphasizing a different melodic line (corresponding respectively to the soprano, alto, and tenor parts). This afforded us an opportunity to evaluate the impact of following an explicit interpretive strategy on the expression of individuality in addition to investigating piece-related effects. Four expressive parameters were analyzed for all performances: articulation (corresponding to the amount of overlap between successive notes, from *staccato* to *legato*), note onset asynchrony (defined as the difference in onset time between events that are notated as synchronous in the score), timing (variations in tempo), and velocity (key press velocity). In line with Stamatatos and Widmer (2005), we extracted these expressive parameters from the MIDI data corresponding to the recordings of the performances. As with organ performance (Gingras, 2008; Gingras et al., 2010), the harpsichord affords no or very little timbre differentiation (excluding registration changes), and dynamic differentiation remains limited (Penttinen, 2006). Thus, most of the expressive features available to harpsichordists, such as articulation, onset asynchrony, and tempo variations, involve the manipulation of timing-related parameters, making the study of expressivity in harpsichord performance ideally suited for the type of MIDI-based quantitative analysis that we propose here.

We used two statistical approaches to investigate expressive individuality in harpsichord performance. The first approach consists in analyzing global piece- or performer-specific trends by examining average expressive tendencies over entire performances, whereas the second approach corresponds to a comparison of expressive profiles at the note-by-note level. Both methods provide complementary information when analyzing expressive patterns in performance (Palmer, 1989; Moelants, 2000). With the first approach, we sought to isolate and quantify the influence of the piece being performed (or the interpretive strategy being followed), as well as the impact of the performer's own stylistic individuality, on the average levels associated with each specific expressive parameter. For instance, this method could be used to determine whether there were significant differences in the mean velocity levels associated with different performers, pieces, or interpretations. One drawback of this approach is that, because it focuses on statistically significant differences observed on mean values representing the average level of an expressive parameter for each performance, it is not suitable for analyzing differences in expressive profiles that are only manifested at the note-by-note level, a problem for which our second approach was better suited. Our aim was twofold with this second approach: first, we sought to determine whether we could detect within-piece concordance among the expressive profiles corresponding to different expressive parameters, when considering performances of the same piece (and similarly when comparing performances following the same interpretive goal in the case of the *Partita*). For instance, we wanted to evaluate whether two performers who display similar articulation profiles when playing the same piece also tend to display similar timing profiles, and whether the reverse is also true for performers who display dissimilar expressive profiles. Second, we examined within-parameter concordance across pieces (or interpretations) when considering profiles associated with a single expressive parameter. For example, we investigated whether two performers who display similar articulation profiles when playing one piece also tend to display similar articulation profiles when playing another piece.

The first approach described here corresponds essentially to an analysis of variance, or more generally to a broad category of statistical methods defined as *general linear models*. Here, because we were interested specifically in isolating the contribution of each individual performer (modeled as a *random effect*) and of each piece or interpretive goal (modeled as a *fixed effect*) to the observed variance for each expressive parameter, we used *linear mixed models* (LMMs) to obtain maximum likelihood (ML) estimates of the “piece” (or “interpretation”) and “performer” effects (Laird and Ware, 1982; Laird et al., 1987; Lindstrom and Bates, 1988). LMMs are a particularly appropriate statistical tool to address these issues because they can fit a variety of covariance structures and allow for the specification of both random intercepts (i.e., fitting individual intercepts for each performer, corresponding to the overall mean values across all pieces for a given expressive parameter), and random slope effects (fitting individual effects associated with each piece for each performer) (West et al., 2007). Although random slope effects are often neglected, Schielzeth and Forstmeier (2009) have shown that ignoring random slope effects tends to overestimate fixed effects in mixed-model designs.

The second approach outlined above is akin to a *similarity analysis* on expressive profiles. Here, we used the correlation between pairs of expressive profiles as a similarity metric. As a normalized and dimensionless similarity metric, the correlation coefficient is appropriate for comparing variables with different units or scales, such as different expressive parameters, and is especially useful for comparing profiles or sequences (Hubert, 1979). Thus, correlation coefficients are among the most effective measures for detecting similarity in gene expression profiles (Yona et al., 2006), a research question which has many parallels with the similarity analysis of expressive profiles in music performance. Unlike the parametric Pearson correlation coefficient, non-parametric correlation coefficients such as Spearman's rho and Kendall's tau are not sensitive to outliers and are less affected by the shape of the statistical distribution of the data, making them more widely applicable as similarity indices. Indeed, a recent study identified Spearman's rho and Kendall's tau as being among the most effective measures for identifying gene coexpression networks (Kumari et al., 2012). Furthermore, non-parametric correlations were shown to be more efficient than parametric measures for detecting stylistic similarity between texts (Popescu and Dinu, 2009).

In contrast to Spearman's rho which is mathematically equivalent to Pearson's coefficient computed on ranks, Kendall's tau is a measure of concordance, corresponding to the probability of agreement on the sign of the difference between pairs of values (Newson, 2002). Therefore, Kendall's tau is especially useful if the direction of the change between two points is more important than the ranking of the absolute values of the points comprising a given sequence or profile, and has been shown to perform better than either Pearson's or Spearman's coefficients when correlating psychiatric symptom ratings (Arndt et al., 1999) and when comparing the rate and direction of change in ecological communities (Huhta, 1979). Because we were specifically interested in the degree of concordance between performers' expressive patterns in the present study, we chose to use Kendall's tau correlation coefficient to assess the pairwise similarity between expressive profiles. These pairwise correlations were then used to generate similarity matrices, calculated separately for each expressive parameter and for each piece (and for each interpretation in the case of the *Partita*). Comparisons were first conducted to assess within-piece concordance between similarity matrices computed for all expressive parameters obtained from a single piece. In a second step, similarity matrices computed for all three pieces on the same expressive parameter were compared to assess the degree of within-parameter concordance between expressive profiles associated with different pieces. The same procedure was then repeated to compare different interpretations of the *Partita*. Lastly, to evaluate the impact of the choice of correlation coefficient on our results, we compared the outcomes of similarity analyses employing Spearman's rho vs. Kendall's tau as a similarity metric.

## Results

### Linear mixed model analyses

#### Comparisons across pieces

**Figure 1:**
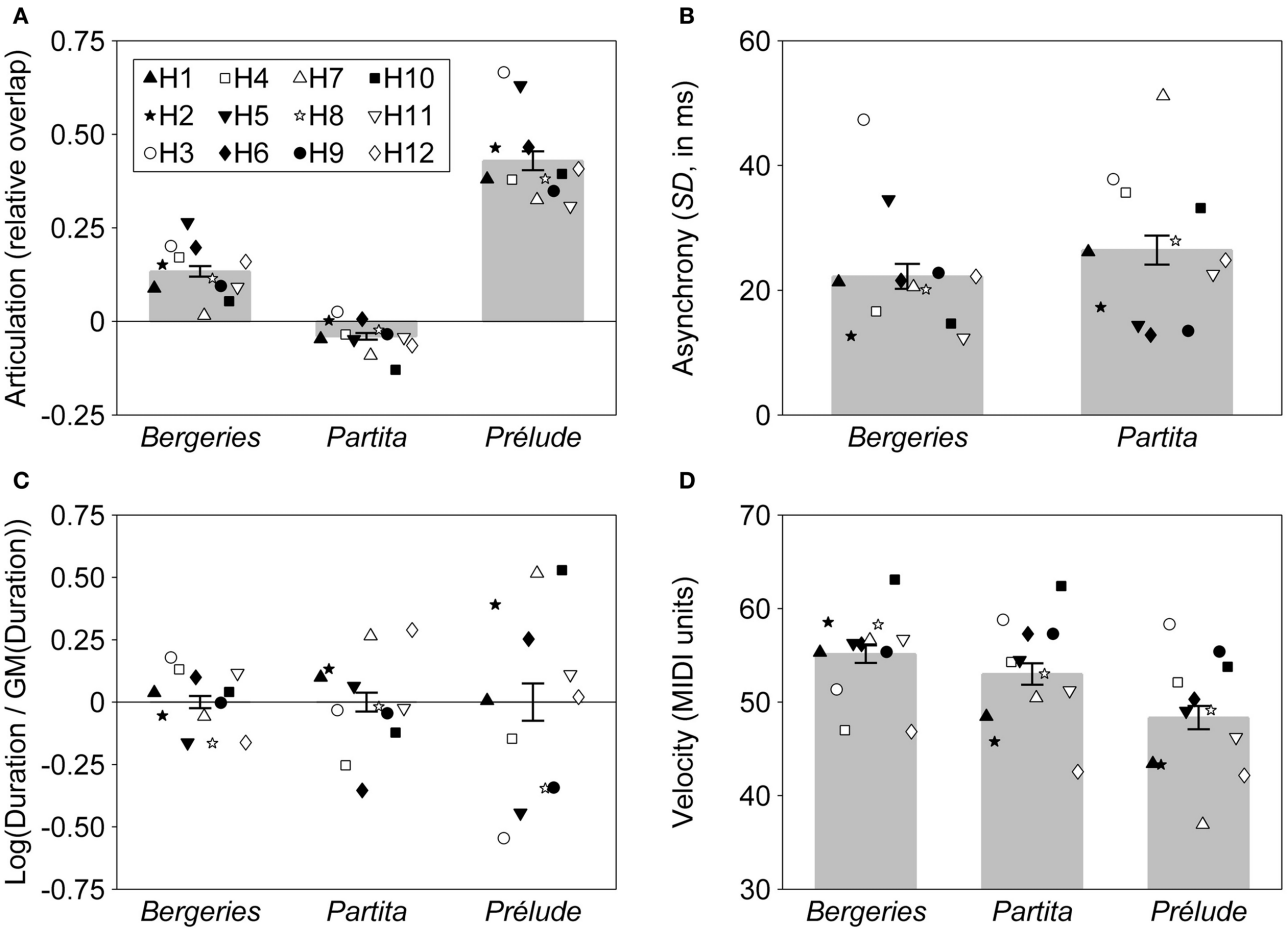
**Mean values for each of the four expressive parameters, for all three pieces.** Each individual harpsichordist (identified as H1, H2, …, H12) is represented by a unique symbol. Each symbol represents the average of two recordings by the same performer. Error bars represent the standard error of the mean. **(A)** Articulation, measured as relative overlap (negative values correspond to a detached articulation and positive values to a *legato* articulation). **(B)** Asynchrony, measured as the standard deviation of onset times for nominally synchronous notes (in milliseconds). **(C)** Timing, measured as the logarithm of the ratio of the duration of the piece to the geometric mean (GM) of the duration of all performances of the same piece. **(D)** Velocity, measured in MIDI units (16–100).

For each of the four expressive parameters (articulation, asynchrony, timing, and velocity), mean values were computed over each performance, separately for each piece (see section Performance Data Analysis in Materials and Methods for computational details). All the analyses of variance presented in this section were conducted on the mean values thus obtained (shown in Figure 1). LMMs were built using the step-up approach (Snijders and Bosker, 1999; Raudenbush and Bryk, 2002), beginning with an unconditional means model with only intercepts for fixed and random effects. For the purpose of conducting comparisons across pieces, we retained only the *Partita* recordings emphasizing the highest melodic line (soprano). Repeated-measures LMMs were used because each piece was recorded twice by each performer, with individual performers (12) treated as random effects and pieces (3) treated as a fixed effect. The potential effect of repetition (comparing the first and second recordings of each piece), as well as the interaction between piece and repetition, were also considered as fixed effects. Note that the models for asynchrony did not include the *Prélude* whose score does not include any note onsets notated as synchronous. Furthermore, the effect of piece was not considered in the case of timing given that durations were zero-centered for each piece to allow for meaningful comparisons across pieces (see section Performance Data Analysis in Materials and Methods).

Fixed effects were first added to the models, followed by random effects. Both random intercepts and random slope effects were considered. At each step, the improvement to the fit of the model was assessed by likelihood tests using ML estimation when comparing models that differed only in the specification of the fixed effects, and restricted maximum likelihood (REML) estimation when comparing models that differed only in the specification of the random effects (Morrell, 1998; Verbeke and Molenberghs, 2000). The following paragraphs outline the model building steps. Detailed tests of significance are only provided for the final models (see Table 1) since all further analyses were conducted on the final models. However, a summary of the *p*-values obtained during the model-building steps is given below where relevant.

**Table 1:** **Linear mixed models comparing across recordings of the three pieces**.

**Expressive**	**Fixed effects**	**Random effects (performer)**		***R*^2^_GLMM_**	
**parameter**					
	**Piece**	**Intercept**	**Piece**	**Marginal**	**Conditional**
		**(overall mean)**	**(slope)**	**(fixed)**	**(fixed and random)**
Articulation	*F*_(2, 22)_ = 223.05, *****p*** < **0.001****	χ^2^(1) = 8.76, *****p*** = **0.003****	χ^2^(1) = 7.75, *****p*** = **0.005****	0.836	0.920
Asynchrony^†^	*F*_(1, 11)_ = 1.02, *p* = 0.335	χ^2^(1) = 0.10, *p* = 0.756	χ^2^(1) = 58.31, *****p*** < **0.001****	0.038	0.426
Timing^*^	N/A	χ^2^(1) = 0.001, *p* = 0.978	χ^2^(1) = 95.59, *****p*** < **0.001****	N/A	0.197
Velocity	*F*_(2, 22)_ = 7.85, *****p*** = **0.003****	χ^2^(1) = 4.82, *****p*** = **0.028****	χ^2^(1) = 78.85, *****p*** < **0.001****	0.210	0.625

The significance of the fixed effect of piece was assessed with Type III F-tests conducted on the final models, whereas the significance of the random intercept and slope effects was assessed with likelihood tests using REML estimation. Statistically significant p-values are indicated in bold. For each expressive parameter, the corresponding marginal and conditional R^2^_GLMM_ values were computed on a random-intercept model that was equivalent to the final model but with the random slope effect excluded (see Nakagawa and Schielzeth, 2013).

† Asynchrony values were not computed for the Prélude, whose score does not include notes that should be played together.

* The fixed effect of piece was not considered for timing, given that all values were zero-centered for each piece to allow for meaningful comparisons across pieces.

In comparison to the baseline model including only intercepts for fixed and random effects, the addition of a fixed effect of piece significantly improved the fit of the models for articulation and velocity (*p* < 0.001 in both cases), but was only marginally significant in the case of asynchrony (*p* = 0.08). The effect of piece was nevertheless included in all three models to facilitate comparisons between models (Cheng et al., 2009). On the other hand, adding the effect of repetition or the interaction between piece and repetition did not improve the fit of the models (all *p*-values > 0.41 for repetition, and all *p*-values > 0.27 for the interaction between piece and repetition). Therefore, the models obtained at the end of this step incorporated a fixed effect of piece (except in the case of timing) and a random intercept.

In a second step, random effects were added. In order to ascertain that random effects, corresponding to individual effects associated with each performer, were significant, we first compared the fit of the models obtained at the end of the first step with equivalent models including only fixed effects (no random intercept). Indeed, models including a random intercept fitted the data significantly better than models incorporating only fixed effects (all *p*-values < 0.05). Subsequently, the inclusion of a random effect of piece was also considered. Adding a random effect of piece improved the fit for all models (all *p*-values < 0.01), leading to our final models, which included a fixed effect of piece, a random intercept, and a random effect of piece (Table 1). Note that in the case of the models for asynchrony and timing, the inclusion of a random piece effect resulted in a non-significant random intercept, suggesting that most of the between-performers variance observed for these two expressive parameters was captured by the random piece effect (we will revisit this point below). Nevertheless, the random intercept was kept in all final models in order to facilitate comparisons between models.

Finally, we sought to directly quantify the variance explained by the fixed (piece) and random (performer) effects in our models. In contrast to traditional general linear models, there is no standard formula for computing the proportion of variance (*R*^2^) explained by the various parameters of a linear mixed model. In this paper, we use a promising approach for estimating *R*^2^ in generalized LMMs (GLMMs, which include LMMs) that was proposed by Nakagawa and Schielzeth (2013). This method can be used to obtain the proportion of variance explained by the fixed effects in a model [defined as “marginal” *R*^2^, or *R*^2^_GLMM_(_*m*_) in Nakagawa and Schielzeth's notation], and the proportion of variance explained by both fixed and random effects [“conditional” *R*^2^, notated as *R*^2^_GLMM (c)_]. The proportion of variance explained by random effects alone can be estimated by comparing both quantities. Note that Nakagawa and Schielzeth's formula does not account for random slope effects (here, random piece effects). However, *R*^2^ values obtained for random-slope models are usually very similar to those obtained for analogous random-intercept models when the same fixed effects are fitted (Snijders and Bosker, 1999). Therefore, we have followed Nakagawa and Schielzeth's (2013) suggestion of computing *R*^2^_GLMM_ values for random-slope models on analogous random-intercept models. The *R*^2^ values reported in Table 1 are thus only an approximation of the *R*^2^ values for the final models, which include a random slope effect.

A comparison of the marginal *R*^2^ values obtained for the different expressive parameters shows that the fixed effect of piece was dominant in the case of articulation, explaining more than 80% of the total variance, suggesting that the overall articulation pattern (detached or *legato*) was mostly a function of the specific piece to be performed, with performer-associated effects playing only a minor role (Figure 1A). On the other hand, the fixed piece effect had only a moderate influence on velocity (Figure 1D) and was negligible in the case of asynchrony (Figure 1B). Random effects (individual differences between performers), which are discussed in greater detail below, played a much larger role for these two expressive parameters than for articulation.

*Post-hoc* tests (pairwise comparisons, all *p*-values Bonferroni-corrected) were conducted for articulation and velocity in order to compare the estimated marginal means for each piece. In the case of articulation, pairwise comparisons showed that the *Prélude* was played significantly more *legato* than both the *Bergeries, t*_(1, 22)_ = 13.15, *p* < 0.001, and the *Partita, t*_(1, 22)_ = 20.89, *p* < 0.001. The *Bergeries* was also played significantly more *legato* than the *Partita, t*_(1, 22)_ = 7.73, *p* < 0.001, giving the following ordering from more detached to more *legato* articulation: *Partita < Bergeries < Prélude* (Figure 1A). Regarding velocity, the *Prélude* was played with significantly less velocity than both the *Partita, t*_(1, 22)_ = 2.66, *p* = 0.043, and the *Bergeries, t*_(1, 22)_ = 3.87, *p* = 0.003, with no significant difference between the latter two (Figure 1D).

Statistically significant random intercepts correspond to a systematic tendency by some performers to display a given expressive feature to a lesser or greater extent than their colleagues, across all pieces. For the four expressive parameters surveyed here, significant random intercepts were only found for articulation and velocity, corresponding to a systematic tendency by some performers to play more detached (or more *legato*), or with a smaller (or greater) velocity than their colleagues, across all pieces (Figures 1A,D). On the contrary, the non-significant random intercepts for the timing and asynchrony models indicate that none of the performers in our sample tended to play significantly slower or faster, or with a lesser or greater degree of asynchrony, than their colleagues when averaging across all pieces (Figures 1B,C).

A significant random piece effect indicates that the effect associated with a given piece is not uniform across all performers, or, in other words, that different performers respond differently to a given piece. Significant random piece effects were found for all four expressive parameters, with large effects in the case of asynchrony, timing, and velocity. The weaker random piece effect for articulation is linked to the strong fixed effect observed for this parameter, which suggests that performers tended to respond more uniformly to piece effects in the case of articulation than for other parameters such as asynchrony and velocity, for which the magnitude of the fixed effect was comparatively smaller.

LMMs allow random effects to be predicted for individual performers (Littell et al., 2006, chapter 8). A summary of the significant intercept and piece random effects at the performer level is provided in Table 2, with individual harpsichordists identified by codes H1 to H12. In line with the results reported previously, no significant random intercepts were found for asynchrony and timing, and only two performers showed significant random piece effects for articulation. Significant random piece effects were especially prevalent for timing, notably in the case of the *Prélude*, suggesting a greater degree of individual variability in the choice of tempi (Figure 1C). Furthermore, we can also see in Table 2 that some performers displayed a greater degree of expressive individuality, as indicated by a large number of significant random effects, than others who showed few or no significant effects (see also Figure 1). For instance, significant effects were associated with performer H3 for all four expressive parameters, but no effects reached significance for performer H1.

**Table 2:** **Individual random effects associated with each performer**.

**Expressive parameter**	**Intercept (overall mean)**	**Piece (slope)**		
		***Bergeries***	***Partita***	***Prélude***
Articulation (*df* = 36)	Detached: H7^*^	n.s.	n.s.	*Legato*: H3^**^, H5^*^
	*Legato*: H3^**^,H5^*^			
Asynchrony^†^ (*df* = 24)	n.s.	More: H3^***^, H5^**^	Less: H5^*^, H6^**^, H9^*^	N/A
			More: H7^***^	
Timing(*df* = 36)	n.s.	Slower: H3^**^, H4^*^, H11^*^	Slower: H2^*^, H7^***^, H12^***^	Slower: H2^***^, H6^***^, H7^***^, H10^***^, H11^*^
		Faster: H5^**^, H8^**^, H12^**^	Faster: H4^***^, H6^***^, H10^*^	Faster: H3^***^, H4^*^, H5^***^, H8^***^, H9^***^
Velocity(*df* = 36)	Less: H12^*^	Less: H3^*^, H4^**^	Less: H2^*^, H12^*^	Less: H7^**^
	More: H10^*^	More: H2^*^		More: H3^**^

Individual performers are identified by codes H1 to H12. The significance of the random intercept and piece effects predicted for each individual performer was assessed using two-tailed t-tests. The denominator degrees of freedom are indicated for each expressive parameter in the leftmost column.

* p < 0.05;

** p < 0.01;

*** p < 0.001; n.s., no significant effect.

† Asynchrony values were not computed for the Prélude, whose score does not include notes that should be played together.

Finally, to control for the fact that all the between-pieces comparisons conducted here employed the interpretation of the *Partita* emphasizing the highest melodic line (soprano), we repeated the LMM analyses described above using the interpretations of the *Partita* emphasizing the alto and tenor parts in turn. We obtained very similar results to those shown in Table 1, both for the *F*-tests on the fixed piece effect and for the likelihood tests on the random intercept and piece effects, with identical outcomes for the significance tests and similar *F* ratios and chi-square values in all cases. This result suggests that the choice of the interpretation of the *Partita* for the purpose of conducting comparisons across pieces had very little bearing on the results of the analyses presented here.

#### Comparisons across interpretations of the partita

Because performers recorded three different interpretations of the *Partita*, we also analyzed the contribution of the interpretive goal and of performers' individual specificities to the variance observed on the mean values for each of the four expressive parameters across interpretations of the *Partita*. Following the procedure described in the preceding section, repeated-measures LMMs were built using the step-up approach, beginning with an unconditional means model with only intercepts for fixed and random effects, treating individual performers (12) as random effects and interpretations (3) as a fixed effect. Once again, repetition (comparing the first and second recordings of each interpretation), as well as the interaction between interpretation and repetition, were considered as fixed effects. Given that the timing comparisons were conducted across interpretations of the same piece in this case, we used the untransformed durations of the performances here (see Performance Data Analysis in Materials and Methods).

In comparison to the baseline model including only intercepts for fixed and random effects, the addition of a fixed effect of interpretation significantly improved the fit of the model for asynchrony (*p* = 0.04) and marginally for articulation (*p* = 0.10), but not for either timing or velocity (both *p*-values > 0.16). The effect of interpretation was nevertheless included in all four models to facilitate comparisons between models. In contrast to what was observed when comparing across pieces, adding a fixed effect of repetition significantly improved the fit of the model for asynchrony (*p* = 0.01), but not for the other parameters (all other *p*-values > 0.19). Again, the effect of repetition was added to all four models. The addition of the interaction between interpretation and repetition did not significantly improve the fit of any model (all *p*-values > 0.11). Thus, the models obtained at the end of this step incorporated fixed effects of interpretation and repetition as well as a random intercept.

Random effects were then examined. We confirmed that models including a random intercept fitted the data significantly better than models incorporating only fixed effects (all *p*-values < 0.001). Subsequently, the inclusion of a random effect of interpretation was also considered. Adding a random effect of interpretation improved the fit for articulation and timing (both *p*-values < 0.01), but not for asynchrony or velocity (both *p*-values > 0.12). The random effect of interpretation was included in all four models. Because a fixed effect of repetition was included in the models, we also considered a random effect of repetition, but its addition did not improve the fit of any models (all *p*-values > 0.12). Hence, our final models included fixed effects of interpretation and repetition, a random intercept, and a random effect of interpretation (Table 3).

**Table 3:** **Linear mixed models comparing across interpretations of the *Partita***.

**Expressive parameter**	**Fixed effects**		**Random effects (performer)**		***R*^2^_**GLMM**_**	
	**Interpretation**	**Repetition**	**Intercept**	**Interpretation**	**Marginal**	**Conditional**
			**(overall mean)**	**(slope)**	**(fixed)**	**(fixed and random)**
Articulation	*F*_(2, 22)_ = 1.32, *p* = 0.287	*F*_(1, 35)_ = 3.25, *p* = 0.080	χ^2^(1) = 28.43, *****p*** < **0.001****	χ^2^(1) = 11.06, *****p*** < **0.001****	0.016	0.823
Asynchrony	*F*_(2, 22)_ = 2.85, *p* = 0.079	*F*_(1, 35)_ = 7.44, *****p*** = **0.010****	χ^2^(1) = 48.81, *****p*** < **0.001****	χ^2^(1) = 1.23, *p* = 0.267	0.018	0.905
Timing	*F*_(2, 22)_ = 0.06, *p* = 0.944	*F*_(1, 35)_ = 0.40, *p* = 0.531	χ^2^(1) = 68.85, *****p*** < **0.001****	χ^2^(1) = 8.24, *****p*** = **0.004****	<0.001	0.970
Velocity	*F*_(2, 22)_ = 1.27, *p* = 0.300	*F*_(1, 35)_ = 0.24, *p* = 0.625	χ^2^(1) = 58.51, *****p*** < **0.001****	χ^2^(1) = 2.41, *p* = 0.120	0.003	0.942

The significance of the fixed effects of interpretation and repetition was assessed with Type III F-tests conducted on the final models, whereas the significance of the random intercept and slope effects was assessed with likelihood tests using REML estimation. Statistically significant p-values are indicated in bold. For each expressive parameter, the corresponding marginal and conditional R^2^_GLMM_ values were computed on a random-intercept model that was equivalent to the final model but with the random slope effect excluded (see Nakagawa and Schielzeth, 2013).

*R*^2^_GLMM_ values were computed following the procedure described in the previous section. Fixed effects explained only a small proportion of the variance, even for the expressive parameters for which these effects were significant or marginally significant, such as articulation and asynchrony. However, the conditional *R*^2^_GLMM_ values were very high, with all four models explaining more than 80% of the variance. The very large proportion of variance explained by random effects for models comparing across interpretations implies that performer-related specificities could account for most of the observed differences in the mean values of the expressive parameters.

**Figure 2:**
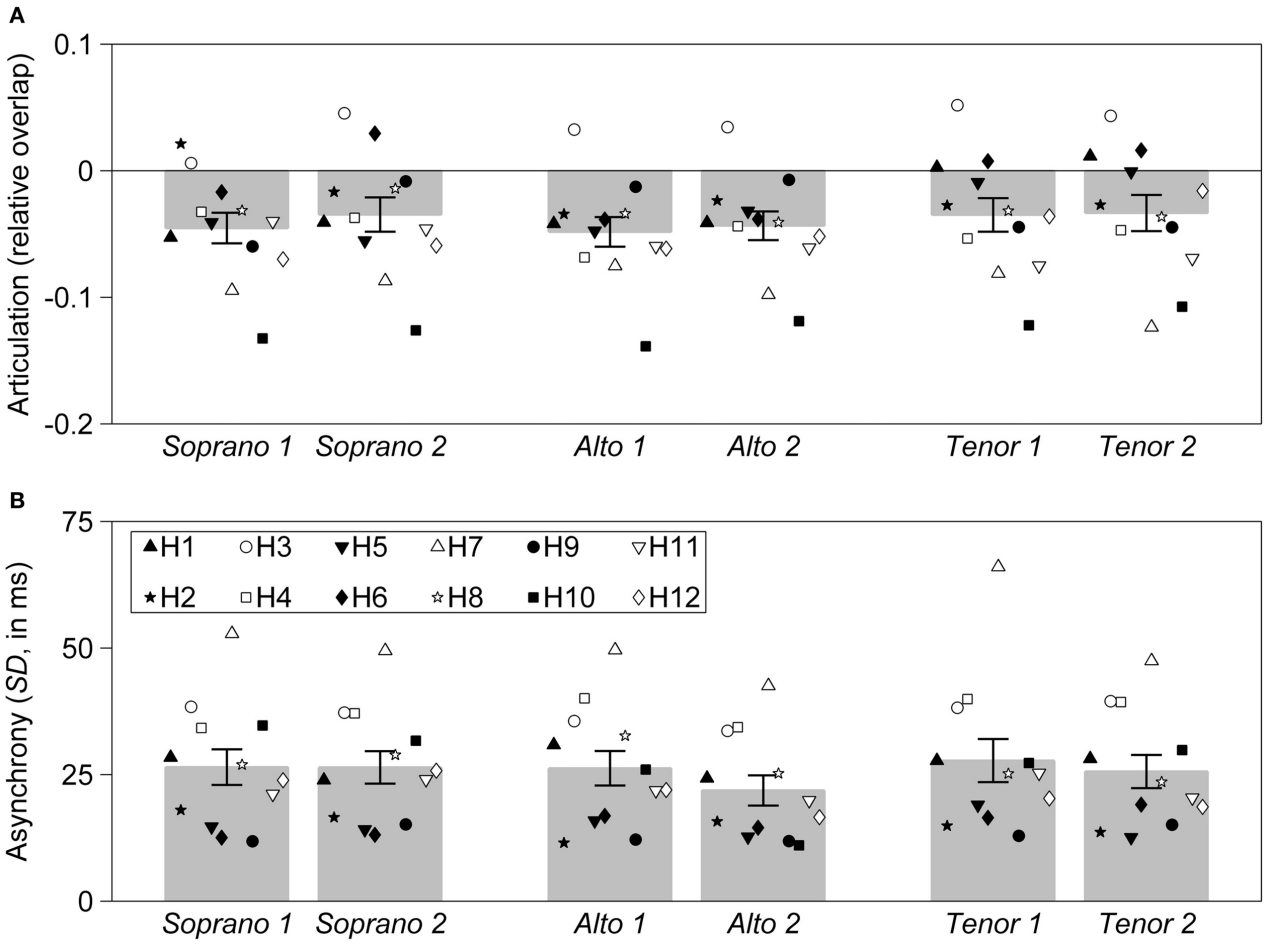
**Mean values for articulation and asynchrony, for all three interpretations of the *Partita***. Each individual harpsichordist (identified as H1, H2, …, H12) is represented by a unique symbol. Each symbol represents a single recording. Three interpretations, each emphasizing a different melodic line (corresponding to the soprano, alto, or tenor part) were recorded. Each interpretation was recorded twice, with successive recordings indicated by the number “1” or “2.” Error bars represent the standard error of the mean. **(A)** Articulation, measured as relative overlap (negative values correspond to a detached articulation and positive values to a *legato* articulation). **(B)** Asynchrony, measured as the standard deviation of onset times for nominally synchronous notes (in milliseconds).

The significant effect of repetition observed in the case of asynchrony corresponded to a tendency by performers to play the second recording of each interpretation with smaller asynchronies than the first (Figure 2B). Similarly, a marginal tendency to play the second recording more *legato* was observed (Figure 2A). To further investigate the effect of repetition in the comparisons across interpretations of the *Partita*, we considered the possibility that the repetition effect was a learning effect, and that performers were still getting accustomed to each interpretation. We thus analyzed the error rates using a GLMM that models the frequency of score errors as a function of the interpretation and the repetition, using a logit (binomial) distribution. This GLMM corresponded to a repeated-measures logistic regression with interpretation and repetition as fixed effects, and random intercept as well as random effect of interpretation, and was thus analogous to the LMMs presented in Table 3. Although error rates were slightly lower for the second repetition (0.69% on average, vs. 0.82% for the first repetition), neither the effect of repetition, *F*_(1, 35)_ = 0.87, *p* = 0.36, nor the effect of interpretation, *F*_(2, 22)_ = 0.49, *p* = 0.62, were close to reaching significance.

Although large statistical effects were associated with the random intercepts for all expressive parameters, significant random interpretation effects were only found for articulation and timing. Random interpretation effects were generally smaller than the effects observed for the random intercepts, as indicated by the relative magnitude of the chi-square values obtained with likelihood tests (Table 3). In line with these results, very few random interpretation effects associated with individual performers were observed. In fact, only one such effect reached significance across all performers and expressive parameters, corresponding to performer H12 playing the “alto” interpretation with a significantly slower tempo. In contrast, a large number of significant random intercepts associated with individual performers were observed. Notably, most performers who exhibited a tendency to play significantly more detached (H7) or more *legato* (H3), or with less (H12) or more (H10) velocity than their colleagues when comparing across pieces (see Table 2), also displayed the same tendencies when comparing across interpretations of the *Partita*. One exception was H5 who showed a significant tendency to play more *legato* across all three pieces, but not across interpretations of the *Partita* (Figure 2A).

#### Discussion

In contrast with the LMMs comparing expressive parameters across pieces, for which important fixed effects were found for articulation and velocity, the proportion of the variance explained by fixed effects was very low for the LMMs comparing interpretations of the *Partita*. This suggests that systematic interpretation-related (or repetition-related) differences between interpretations emphasizing different melodic lines were, for the most part, relatively unimportant when comparing mean values computed on the entire performances. To be sure, this does not imply that there were no significant differences between these interpretations, but analyzing these differences requires a finer approach which involves considering each melodic line in isolation (Gingras et al., 2009). On the other hand, random effects explained a much larger proportion of the variance for the LMMs comparing across interpretations of the *Partita* than for the LMMs comparing across pieces (even though these random effects were non-negligible when accounting for the variance in asynchrony, timing, or velocity across pieces). This result indicates that individual specificities tended to dominate when considering interpretations of the same piece, but were relatively less important when examining different pieces.

The significant effects associated with repetition (i.e., comparing the first and second recordings) in the LMMs on the interpretations of the *Partita* were somewhat unexpected, because repetition was not a significant factor in any of the LMMs modeling expressive parameters across pieces. Adding repetition as a fixed effect to these LMMs did not increase the *R*^2^_GLMM_ values for any of the models. The overall low error rates, as well as the absence of a significant difference in error rates between repetitions or interpretations, suggest that performers were comfortable with each interpretation at the time of recording and do not argue in favor of a learning effect. Nevertheless, it is possible that changing between interpretations of the same piece during the recording session demanded more flexibility on the part of the performers than changing from one piece to another. This may explain why asynchronies were slightly but significantly smaller, and articulations slightly more *legato* (albeit with small effect sizes in both cases), on the second recording of each interpretation as performers were adjusting to the character of each interpretation.

Whereas the magnitude of the random piece effects was generally larger than that of the random intercept effects when comparing across pieces (see Table 1), the opposite was observed when comparing across interpretations of the *Partita* (see Table 3). This suggests that, whereas individual performers exhibited markedly different responses to the three pieces, individual responses to the three interpretations of the *Partita* were not as differentiated. On the other hand, performers who tended to play consistently more *legato*, or with a faster tempo, tended to do so for all three interpretations of the *Partita* (as indicated by the large random intercept effects reported in Table 3), whereas this performer-specific consistency was somewhat less pronounced when comparing across pieces (as indicated by the small to moderate random intercept effects shown in Table 1).

### Similarity analyses on expressive profiles

#### Comparisons across pieces

Kendall's tau correlation coefficients were calculated between the expressive profiles of all pairs of performers, separately for each parameter and for each piece. For the purpose of conducting comparisons across pieces, we retained only the *Partita* recordings emphasizing the highest melodic line (soprano). To avoid pseudo-replication, correlation coefficients were computed on the expressive profiles corresponding to the average of the two performances recorded by each performer for each piece (note that very similar results were obtained by averaging the correlations obtained on each of the two performances instead of computing the correlations on the averaged profiles). Correlation coefficients were computed on a note-by-note basis in the case of articulation and velocity, and on an event-by-event basis in the case of timing and asynchrony. Similarity matrices were then generated by computing all possible pairwise Kendall's taus between the 12 performers' note-by-note (or event-by-event) expressive profiles, separately for each parameter and for each piece. Eleven 12 × 12 similarity matrices were obtained, four each for the *Bergeries* and the *Partita* (one for each expressive parameter), and three for the *Prélude* for which no asynchrony patterns were extant. All correlation coefficients were positive, indicating a higher-than-chance concordance between expressive profiles (the statistical significance of each coefficient is not reported here due to the very large number of correlations, and because the aim of this analysis was not to test the significance of each pairwise correlation but to examine the global concordance between similarity matrices).

Two series of comparisons were conducted between the similarity matrices thus obtained. First, to test for within-piece profile concordance across expressive parameters, we assessed the degree of congruence between the groups of similarity matrices corresponding to all expressive parameters analyzed for a single piece. Second, to test for within-parameter profile concordance across pieces, we assessed the degree of congruence between the groups of similarity matrices corresponding to a single expressive parameter analyzed over all pieces.

To control for familywise error rates, the CADM (“Congruence among distance matrices”) test (Legendre and Lapointe, 2004), which detects congruence in a group of matrices, was first applied to each group of similarity matrices that was tested separately. If the chi-square statistic obtained by the CADM test was significant (as determined by a permutation test), indicating congruence in a group of matrices, *post-hoc* tests were conducted to identify the matrix (or matrices) which explained this association, following Legendre and Lapointe (2004). The Bonferroni-Holm correction (Holm, 1979), a sequential procedure which is less conservative than the classic Bonferroni correction, was applied to the *p*-values thus obtained. Lastly, the Mantel test, a non-parametric permutation test which evaluates the degree of association between two matrices (Mantel, 1967; Legendre and Legendre, 1998) and is applicable to either distance matrices or similarity matrices (Dietz, 1983), was used to determine the pairwise rank correlation (Spearman's rho) between the similarity matrix (or matrices) identified as significantly congruent in the *post-hoc* procedure and other matrices in the group. Note that, by design, both the CADM and Mantel tests ignore the main diagonal of the matrices, meaning that all the comparisons presented here were strictly conducted between expressive profiles corresponding to different performers. The number of degrees of freedom does not affect the probability values obtained by permutation tests (McArdle and Anderson, 2001) and is not reported for the CADM and Mantel tests (see Legendre, 2000).

**Figure 3:**
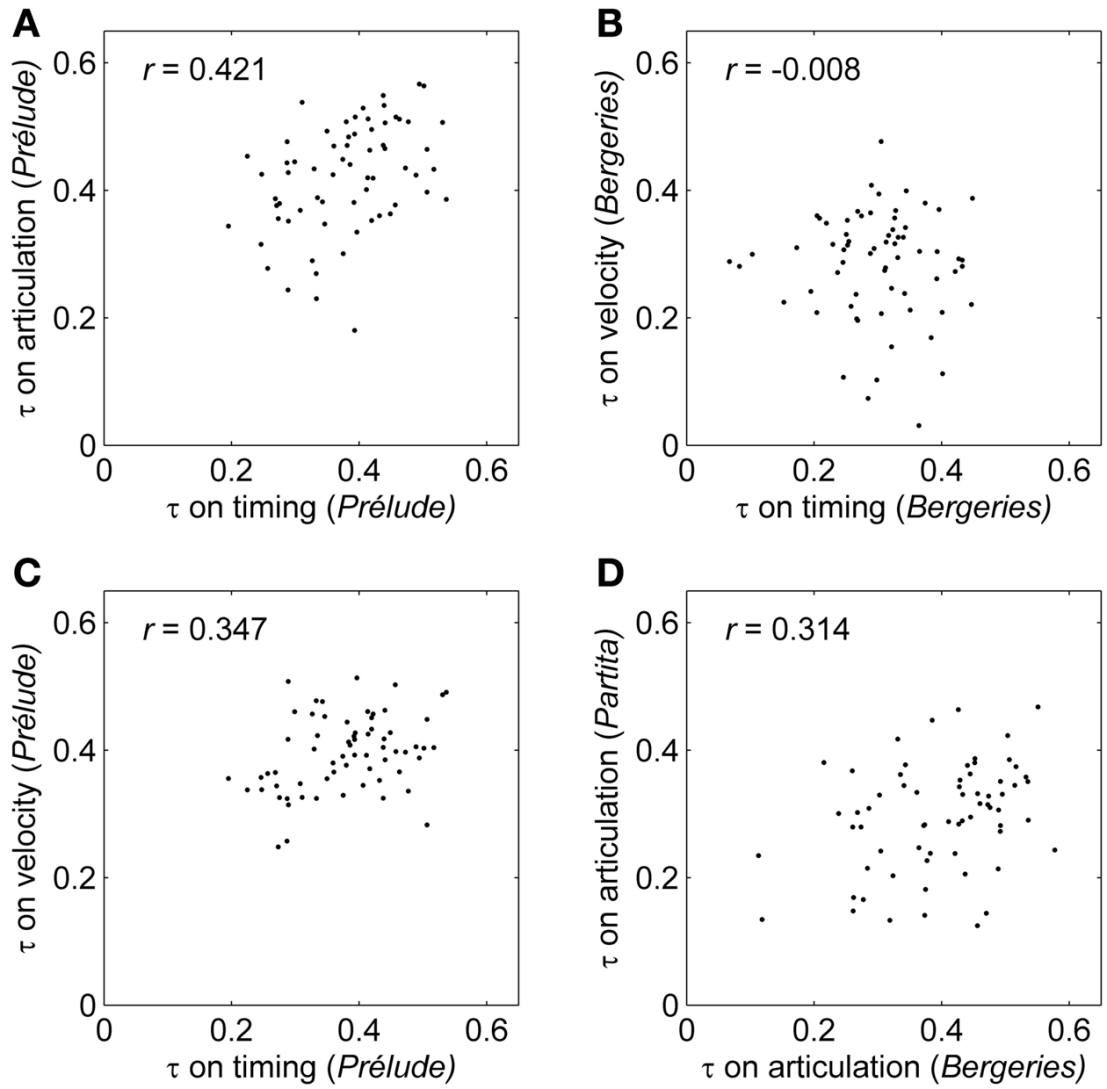
**Concordance between the pairwise correlations computed on expressive profiles**. τ: Kendall's tau correlation coefficient computed on expressive profiles between pairs of performers. *r*: Mantel correlation coefficient between similarity matrices. Each dot corresponds to the pairwise correlation between two performers. **(A)** Timing and articulation pairwise correlations for the *Prélude*. **(B)** An example of a non-significant association between the timing and velocity pairwise correlations for the *Bergeries*. **(C)** Timing and velocity pairwise correlations for the *Prélude*. **(D)** Pairwise correlations on the articulation profiles for the *Bergeries* and the *Partita*.

CADM tests were first conducted to assess the within-piece congruence between the similarity matrices corresponding to the four expressive parameters (only three in the case of the *Prélude*), separately for each piece. A significant association was detected for the *Prélude*, χ^2^ = 94.95, *p* = 0.020. *Post-hoc* tests revealed that the timing similarity matrix was significantly congruent with at least one other matrix in the group (Bonferroni-Holm corrected *p*-value < 0.001). Mantel tests showed a significant correlation between the matrices for timing and articulation (*r* = 0.421, *p* = 0.009), indicating that the magnitude of the pairwise correlations computed between all pairs of performers on the timing profiles was positively correlated with the magnitude of the corresponding pairwise correlations computed on the articulation profiles (Figure 3A; see also Figure 3B for a visual representation of a non-significant association between the timing and velocity pairwise correlations for the *Bergeries*). In other words, there was a significant tendency for performers with concordant timing patterns to show concordant articulation patterns. The correlation between the similarity matrices for timing and velocity also reached significance (*r* = 0.347, *p* = 0.032), corresponding to a tendency for performers with concordant timing profiles to also display concordant velocity profiles (Figure 3C). Furthermore, the CADM tests were also marginally significant for the *Bergeries* (χ^2^ = 84.40, *p* = 0.076) and the *Partita* (χ^2^ = 85.31, *p* = 0.085), suggesting weak or partial congruence in both cases (no *post-hoc* tests were conducted here since the tests did not reach significance).

Second, CADM tests were conducted to assess the within-parameter congruence among the similarity matrices based on a single expressive parameter across all pieces, separately for each of the four parameters. A significant association was detected for articulation (χ^2^ = 98.33, *p* = 0.010) and for timing (χ^2^ = 88.29, *p* = 0.041), but not for asynchrony or velocity (both *p*-values > 0.27). For articulation, *post-hoc* tests revealed that the articulation similarity matrix for the *Partita* was congruent with at least one other matrix (Bonferroni-Holm corrected *p*-value = 0.040). However, the corrected *p*-values for the matrices corresponding to the *Bergeries* and the *Prélude* were both marginally significant, suggesting that the articulation similarity matrices for all three pieces were at least partially congruent. Mantel tests showed a significant correlation between the matrices for the *Bergeries* and the *Partita* (*r* = 0.314, *p* = 0.038), indicating that the magnitude of the pairwise correlations computed between the *Bergeries* articulation profiles for all pairs of performers was correlated with the magnitude of the corresponding pairwise correlations computed on articulation profiles for the *Partita* (Figure 3D). A marginally significant correlation was also observed between the articulation similarity matrices for the *Partita* and the *Prélude* (*r* = 0.241, *p* = 0.072). For timing, *post-hoc* tests revealed that the timing similarity matrix for the *Prélude* was congruent with at least one other matrix (Bonferroni-Holm corrected *p*-value = 0.045), and Mantel tests showed a significant correlation between the timing matrices for the *Prélude* and the *Partita* (*r* = 0.376, *p* = 0.033).

Finally, to control for the fact that the comparisons across pieces employed the interpretation of the *Partita* emphasizing the highest melodic line (soprano), we repeated these analyses using the interpretations of the *Partita* which emphasized the alto and tenor parts, respectively. We obtained similar results to those described in the previous paragraph, with identical outcomes for the CADM tests in practically all cases. Exceptions were the CADM test on asynchrony, which was marginally significant with the alto interpretation (χ^2^ = 82.22, *p* = 0.092) but not with other interpretations (all other *p*-values > 0.27), and the CADM test on timing, which reached significance with either the soprano or tenor interpretations (both *p*-values < 0.05), but was only marginally significant with the alto interpretation (χ^2^ = 83.28, *p* = 0.077). This suggests that the choice of the interpretation of the *Partita* for the purpose of conducting comparisons across pieces had only a minor influence on the outcome of the similarity analyses.

#### Comparisons across interpretations of the partita

Following the procedure described above, similarity matrices were generated by computing all possible pairwise Kendall's taus between the 12 performers' note-by-note (or event-by-event) expressive profiles, separately for each parameter and for each interpretation of the *Partita*. With very few exceptions for which slightly negative values were obtained (corresponding to 3 out of 792 pairwise correlations), all Kendall's taus were positive, indicating a higher-than-chance concordance between expressive profiles. Twelve 12 × 12 similarity matrices were obtained, for each of the four expressive parameters and each of the three interpretations.

CADM tests were first conducted to assess the within-interpretation congruence among the similarity matrices corresponding to the four expressive parameters, separately for each interpretation. As reported in the previous section, a marginal tendency was found for the soprano interpretation (χ^2^ = 85.31, *p* = 0.085). Additionally, a significant association was detected for the alto (χ^2^ = 97.22, *p* = 0.026) and tenor (χ^2^ = 102.01, *p* = 0.005) interpretations. In the case of the alto interpretation, *post-hoc* tests revealed that the asynchrony and timing similarity matrices were congruent with at least one other matrix (both Bonferroni-Holm corrected *p*-values < 0.01). Mantel tests showed a significant correlation between the asynchrony and timing matrices (*r* = 0.558, *p* = 0.001) and between the velocity and timing matrices (*r* = 0.373, *p* = 0.009). For the tenor interpretation, *post-hoc* tests indicated that the timing matrix was congruent with at least one other matrix (Bonferroni-Holm corrected *p*-value < 0.001). Mantel tests showed that the timing matrix was significantly correlated with the articulation (*r* = 0.409, *p* = 0.022), asynchrony (*r* = 0.391, *p* = 0.007), and velocity (*r* = 0.283, *p* = 0.022) matrices.

CADM tests were then conducted to assess the within-parameter congruence among the similarity matrices based on a single expressive parameter across all interpretations, separately for each of the four parameters. The CADM tests were highly significant for all parameters (all χ^2^ >125, all *p*-values < 0.001). *Post-hoc* tests revealed that all matrices corresponding to the same expressive parameter were congruent with each other (all Bonferroni-Holm corrected *p*-values < 0.01). Similarly, Mantel tests indicated that all pairwise correlations conducted between similarity matrices corresponding to the same parameter were significant (all *r* > 0.39, all *p*-values < 0.01).

#### Comparison between Kendall's tau and Spearman's rho

In order to evaluate whether the choice of non-parametric correlation coefficient affected the outcome of the similarity analyses reported in the preceding sections, we repeated all analyses using Spearman's rho correlation coefficient instead of Kendall's tau. The CADM tests conducted on the similarity matrices generated using Spearman's rho coefficients yielded chi-square and *p*-values very similar to those obtained on the corresponding matrices generated using Kendall's tau, with identical outcomes for the significance tests in all cases except for the within-piece, across-parameters congruence for the *Prélude* which was marginally significant with Spearman's rho (χ^2^ = 85.35, *p* = 0.074) but reached significance with Kendall's tau (χ^2^ = 94.95, *p* = 0.020). Overall, the comparable results obtained with both correlation coefficients suggest that our approach is robust to the type of non-parametric correlation used in the similarity analysis.

#### Discussion

The CADM tests conducted to assess within-piece, or within-interpretation, congruence across all expressive parameters were at least marginally significant for all pieces and interpretations analyzed here, suggesting that this type of concordance across different parameters is not uncommon. For the CADM tests that reached significance (for the *Prélude* and for the alto and tenor interpretations of the *Partita*), *post-hoc* tests showed that, in all cases, the timing similarity matrix was shown to be significantly congruent with at least one other matrix. This indicates that the magnitude of the correlations between the timing profiles of different performers tended to be positively associated with the magnitude of the correlations between the expressive profiles computed on at least one other expressive parameter, suggesting that timing profiles seem to play a central role in the within-piece or within-interpretation congruences observed here.

CADM tests conducted to assess within-parameter congruence across pieces or interpretations revealed much higher congruences across interpretations of the *Partita* than across pieces, a result which is probably expected given that different interpretations of the same piece are likely to be much more similar to each other than performances of different pieces. Indeed, all Mantel correlations between the similarity matrices corresponding to the same expressive parameter were highly significant for all four parameters when comparing across interpretations of the *Partita* (note that Mantel *r* values are often comparatively small even when significant, see Dutilleul et al., 2000). On the other hand, CADM tests revealed significant congruences across pieces only for timing and articulation. These findings suggest that while it is very likely that performers who display concordant profiles for one interpretation of a piece will also display concordant profiles for a different interpretation of the same piece when considering the same expressive parameter, this is not as likely when comparing across pieces, and was only observed on some expressive parameters.

Finally, comparable results were obtained with either Kendall's tau or Spearman's rho as a measure of pairwise similarity between expressive profiles, indicating that the approach presented here is not dependent on a particular type of non-parametric correlation coefficient. Nevertheless, Kendall's tau remains a more general measure of concordance in our view, for the reasons detailed in the Introduction, and is therefore more suitable than Spearman's rho for comparing expressive profiles across pieces (or interpretations) and parameters.

## General discussion

In this article, we sought to disentangle performer- and piece-specific influences on expressive strategies in harpsichord performance, by pursuing two lines of inquiry, one based on an analysis of the proportion of variance in the mean values for each expressive parameter explained by performer and piece (or interpretation) effects, and a second one on a similarity analysis of note-by-note expressive profiles. These analyses were conducted on a dataset of recordings of three pieces representative of the harpsichord repertoire made by 12 professional performers and focused on four expressive parameters: articulation, asynchrony, timing, and velocity.

The first approach used LMMs to show that piece-specific influences explained a large proportion of the variance in the mean values for some expressive parameters such as articulation (and to a lesser extent velocity), whereas analogous analyses on the different interpretations of the *Partita* showed only negligible interpretation-specific effects. On the other hand, individual differences explained a much larger proportion of the variance for the LMMs comparing across interpretations of the *Partita* than for those comparing across pieces, indicating that performer-specific influences were prevalent in the former case. These individual differences can be sorted into two categories: (1) piece- or interpretation-related differences between performers (corresponding to a significant random slope in a linear model) and (2) global differences between performers across all pieces or interpretations (corresponding to a significant random intercept). The former were observed on all expressive parameters when comparing across pieces but were generally less important when comparing across different interpretations of the same piece, whereas the latter only reached significance for articulation and velocity when comparing across pieces but amounted to large effects when comparing across interpretations. The fact that some individual performers consistently deviated from the mean values for some expressive parameters, both across different pieces and across interpretations of the same piece, suggests that global, undifferentiated expressivity measures computed over an entire performance, such as the mean overlap or the average key velocity, may in some cases constitute valid markers of artistic individuality that reliably characterize a performer's playing style. Finally, these analyses also revealed important differences in the degree of individuality expressed by performers, with some harpsichordists exhibiting statistically significant individual random intercept or slope effects for several expressive parameters, and others only for a few or none.

The second approach used permutation tests on similarity matrices generated from pairwise Kendall's tau correlations between note-by-note (or event-by-event) expressive profiles to show that, when examining profiles associated with different expressive parameters but all corresponding to the same piece (or same interpretation), we observed in all cases a significant effect, or at least marginally significant tendency, for the degree of concordance between the profiles of different performers for one expressive parameter to be positively correlated with the degree of concordance between their profiles on at least one other expressive parameter. Moreover, we observed that, when comparing profiles associated with the same expressive parameter but corresponding to different interpretations of the *Partita*, there was a significant tendency, for all four expressive parameters, for performers with concordant profiles in one interpretation to also display concordant profiles in another interpretation. This was the case only for articulation and timing when comparing across pieces. These findings have several implications. First, the fact that concordance between different performers in one expressive parameter also tends to be associated with concordance in a different parameter suggests that parameters cannot always be considered in isolation, and that a type of interpretive concinnity can manifest itself across expressive parameters. Although this type of interaction between expressive devices has been reported previously, for instance between tempo and loudness curves (Widmer and Goebl, 2004) or between asynchrony (melody lead) and velocity (Repp, 1996a; but see Goebl, 2001), it had not, to our knowledge, been demonstrated across different pieces or between different performers. Second, the within-parameter, between-pieces congruence observed for articulation and timing indicates that performers who use similar articulation (or timing) patterns in one piece also tend to display similar articulation (or timing) profiles in another piece. This suggests that, at least for these expressive parameters, interpretive choices can transcend pieces and maybe even compositional genres. Lastly, and more generally, these observations point to the existence of expressive meta-strategies encompassing several expressive parameters and that can manifest themselves either across interpretations or even different pieces.

The fact that a significant within-parameter, between-pieces concordance was only observed for articulation and timing may be related to the important role played by these expressive devices in harpsichord performance (Gingras et al., 2009). In particular, the importance of articulation in Baroque music has been noted elsewhere (Rosenblum, 1997; Lawson and Stowell, 1999). In contrast, the relevance of note-by-note velocity patterns is presumably de-emphasized, at least to some extent, due to the limited dynamic differentiation available on the harpsichord (Penttinen, 2006), and it is not clear that note-by-note variations in velocity are intentionally employed for expressive purposes by harpsichordists. Although local tempo variations are generally considered to be intimately related to the formal structure of the pieces under consideration (Todd, 1985; Repp, 1992), individual performers may interpret formal structures in idiosyncratic ways that are consistent across different pieces, so that performers who agree in their timing profiles for one piece tend to agree for a different piece. Hence, the significant between-pieces congruence observed for timing suggests that, for instance, performers using *rallentando* (a gradual slowing down) in similar places in the *Partita* might be expected to also have comparable tempo variation profiles in the *Bergeries*, whereas performers with different profiles for the former piece would also be expected to exhibit less concordance in their profiles for the latter piece. Note-by-note articulation patterns are, presumably, not tightly linked with specific formal structures, but likely correspond to piece-independent patterns that are characteristic of an individual performer's playing style. Perhaps paradoxically, the between-pieces concordance in note-by-note articulation patterns, which suggests the existence of a performer-related specificity that transcends pieces, is contrasted with a very strong piece-specific effect on the global articulation trends (see Table 1). Clearly, more research, using a greater number of pieces and possibly involving comparisons across instruments and repertoires, is necessary not only to elucidate the issues outlined here, but also to investigate more comprehensively the nature and prevalence of the expressive meta-strategies that the present research has uncovered.

A particularity of the experimental design used here is that performers were asked to emphasize a specific melodic line in the *Partita* (although they were free to choose whichever expressive strategy they desired to achieve that aim), whereas they were simply invited to play as if in a recital setting for the other pieces. Although the instruction to emphasize a melodic line would likely have affected the performers' expressive choices, thus potentially biasing our results, we do not believe this to be a major concern here. First, we retained only the *Partita* recordings emphasizing the highest melodic line (soprano), which is probably closest to a natural interpretation (Palmer and Holleran, 1994; Palmer, 1996; Goebl, 2001), for the comparisons across pieces. In any case, very similar results were obtained when substituting the soprano interpretation with the alto or tenor interpretations, both for the LMMs and for the similarity analyses, suggesting that the choice of interpretation had only a minimal impact on our results. Second, the results do not show any evidence that the performers were unable to fully express their individuality in performances of the *Partita*. In that regard, it is especially relevant to note that significant congruences were observed between the timing profiles for the *Prélude* and the *Partita*, as well as for the note-by-note articulation patterns between the *Partita* and the *Bergeries* (with a marginally significant congruence between the *Partita* and the *Prélude*). This indicates that the concordance in timing or articulation patterns between individual performers was preserved between the *Partita* and other pieces, even though the performance instructions were different, which is a noteworthy result in itself.

Besides the dichotomy between measured and unmeasured pieces in our sample, it is likely that other stylistic features, related for instance to the date of composition, the compositional genre, the meter (the *Bergeries* is written in 6/8, whereas the *Partita* follows a 4/4 meter) or the texture (the *Partita* is written in a much more polyphonic style than the other pieces) played a role in the performers' choice of expressive strategies. Our analysis did not account for these aspects, but further research may address them more directly by examining a greater number of pieces and perhaps more explicitly adopting a musicological perspective. Other expressive strategies relating to tempo and meter, such as the use of *notes inégales* (in which some notes with equal written time values are performed with unequal durations, usually as alternating long and short) and other types of durational contrasts (Fabian and Schubert, 2010; Moelants, 2011), or metrical emphasis (strong vs. weak beats), could also be explored in greater depth.

In conclusion, this study highlighted the usefulness of LMMs, and more generally mixed models employing likelihood estimation, in quantifying piece-, interpretation-, and performer-specific influences on expressive choices, as well as the relevance of similarity analyses, based on methods found more commonly in biological sciences (such as the CADM and Mantel tests), to the comparison of expressive profiles across pieces (or interpretations) and expressive parameters. Notably, because our methodology for the similarity analysis relies entirely on non-parametric tests, it is potentially broadly applicable and could likely be generalized to the study of other expressive parameters or even perceptual response profiles. Our findings constitute a significant addition to the literature on individuality in music performance, especially given that very few studies compared the same performers across different pieces or interpretations of the same piece. Finally, the combination of the type of analysis proposed here with empirical studies examining the perception of individuality in music performance (Gingras et al., 2011; Koren and Gingras, 2011) could conceivably prove to be a very fruitful and synergistic endeavor in the investigation of artistic individuality.

## Materials and methods

### Participants

Twelve professional harpsichordists, five female and seven male, from the Montreal (Canada) area were invited to participate in the experiment. Their average age was 39 years (range: 21–61 years). They had played the harpsichord for a mean duration of 22 years (range: 6–40). Seven of them had previously won prizes in regional, national, or international harpsichord competitions. Ten reported being right-handed, one left-handed, and one ambidextrous. All harpsichordists signed a consent form and received financial compensation for their participation in the study, which was approved and reviewed by the Research Ethics Board of McGill University (Montreal, Canada).

### Procedure

For the *Prélude* and the *Bergeries*, performers received no instructions besides playing the pieces as if in a “recital setting.” Each piece was recorded twice. In the case of the *Partita*, performers were instructed to play three versions, each emphasizing a different voice (respectively, the soprano, alto, and tenor parts). Each interpretation was recorded twice, for a total of six recordings per performer. The order of the instructions was randomized according to a Latin square design. Performers were given 20 min to practice before recording the pieces (the scores of the *Prélude* and of the *Bergeries* were given to the performers 4–6 weeks before the recording session). The entire recording session lasted ~1 h.

Performances took place in an acoustically treated studio, on an Italian-style Bigaud harpsichord (Heugel, Paris, France) with two 8-foot stops. Only the back stop was used for the experiment. This harpsichord was equipped with a MIDI console, allowing precise measurement of performance parameters. MIDI velocities were estimated by a mechanical double contact located underneath the keys and from which the travel time of the keys was measured, with a high velocity corresponding to a shorter travel time (faster attack). MIDI velocity values for each note event were coded in a range between 16 (slowest) and 100 (fastest). The measured velocities were calibrated separately for each key by authors Bruno Gingras and Pierre-Yves Asselin.

The audio signal was recorded through two omnidirectional microphones MKH 8020 (Sennheiser GmbH, Wedemark Wennebostel, Germany). The microphones were located 1 m above the resonance board and were placed 25 cm apart. The audio and MIDI signals were sent to a PC computer through an RME Fireface audio interface (Audio AG, Haimhausen, Germany). Audio and MIDI data were then recorded using Cakewalk's SONAR software (Cakewalk, Inc., Boston, MA, USA) and stored on a hard disk.

### Performance data analysis

Performances were matched to the scores of the pieces using an algorithm developed by the authors, which has been shown to be suitable for ornamented harpsichord pieces (Gingras and McAdams, 2011). To ensure that the excerpts from all three pieces were of comparable duration, only the first part of the rondo from the *Bergeries* was used in all subsequent analyses. The excerpt from the *Bergeries* comprised 281 notes, whereas the *Partita* contained 153 notes, and the *Prélude* 140 notes. The average duration (from first to last onset) was 54.2 s for the *Bergeries* excerpt (range: 47.1–61.6 s), 36.8 s for the *Partita* (range: 28.3–47.5 s), and 59.9 s for the *Prélude* (range: 39.1–84.2 s). These durations corresponded to the following tempi: for the *Bergeries*, the mean tempo was 107.0 beats per minute (bpm), ranging from 93.5 to 122.3 bpm, with the beat corresponding to an eighth note (6/8 meter); for the *Partita*, the mean tempo was 52.9 bpm (range: 40.4–67.7 bpm), with the beat corresponding to a quarter note (4/4 meter); for the *Prélude*, the mean tempo was 149.2 bpm (range: 99.8–214.7 bpm), with each note onset counted as a “beat” in the absence of a notated rhythmic structure.

The mean error rates per performance, defined as the proportion of wrong notes or missing notes relative to the total number of score notes, were as follows: for the *Bergeries*, 0.37% (range: 0–1.42%); for the *Partita*, 0.82% (range: 0–2.61%); and for the *Prélude*, 0.54% (range: 0–2.14%). These low error rates are comparable to the rates reported by Repp (1996b) and Goebl (2001) in studies on professional piano performance, suggesting that the performance data collected for the current study were of suitable quality for assessing individual expressive profiles in professional harpsichord performance.

Four expressive parameters were analyzed for each performance: articulation, note onset asynchrony, timing, and velocity. Articulation refers to the amount of overlap between two consecutive note events *n*_*i*_ and *n*_*j*_ belonging to the same melodic line or voice. A *legato* articulation corresponds to a positive overlap (when the offset of note *n*_*i*_ occurs after the onset of note *n*_*j*_), whereas a detached or staccato articulation corresponds to a negative overlap. Here, the onset of a note is defined as the time at which the corresponding key is pressed (as measured by the MIDI system) and its offset corresponds to the time at which the key is released. Because the amount of overlap varies with tempo (Repp, 1995), we chose to use the overlap ratio, defined as the ratio of the overlap between two consecutive note events and the inter-onset interval between these notes, as a measure of articulation (Bresin and Battel, 2000).

Note onset asynchrony is defined as the difference in onset time between note onsets that are notated in the musical score as synchronous (Palmer, 1989). Several measures of onset asynchrony have been constructed. Rasch (1979) proposed to use the root mean square, or standard deviation of the onset times of nominally simultaneous notes. We chose to use this measure here. Onset asynchrony values were not computed in the case of the *Prélude*, whose score does not include nominally synchronous notes.

To analyze expressive timing, tempo values were computed from the inter-onset interval between consecutive note onset events. To allow for meaningful comparisons across pieces for the LMM analysis, the logarithm in base two of the total duration of the piece (defined as the time interval between the first onset and the last onset of the piece, see Moelants, 2000) divided by the geometric mean of the total duration across all performances, was used (Wagner, 1974; see also Repp, 1992). This procedure yields a tempo valuation that is centered and scaled for each piece, with a value of 1 corresponding to a tempo that is twice as slow (duration twice as long) as the mean tempo for all performances of the piece, and a value of −1 corresponding to a tempo that is twice as fast (duration twice as short). Untransformed durations were used for the LMMs analyzing the different interpretations of the *Partita*.

For the event-by-event timing profiles, local tempo values were obtained for each note onset event *e* by computing the logarithm of the ratio of the duration (inter-onset interval) of *e* to its expected duration (i.e., the duration obtained by dividing the notated duration of *e* by the notated duration of the entire piece, corresponding to a “deadpan” or mechanical performance with an invariant tempo) was used. In this case, a local tempo value of 0 for *e* corresponds to the mean tempo of the performance (indicating no local deviation from the mean tempo), whereas a value of 1 corresponds to a local tempo that is twice as slow as the mean tempo, and a value of −1 to a tempo that is twice as fast.

Lastly, in the case of velocity, the raw MIDI velocity values associated with the key press corresponding to each note onset (see Procedure) were used for the analysis.

### Statistical analysis

LMMs were fitted using the PROC MIXED function in SAS 9.0 (SAS Institute, Cary, NC, USA) (Singer, 1998; Littell et al., 2006). All models were fitted using a variance components (VC) covariance matrix, which is the default covariance matrix structure for LMMs both in PROC MIXED and in the analogous MIXED procedure in SPSS 19.0 (SPSS Inc., Chicago, IL, USA). Analogous models were also fitted with the unstructured and compound symmetry covariance structures, but these models either did not converge or yielded worse fits than equivalent LMMs fitted with the default VC structure. Although all reported analyses were conducted in SAS, we verified that equivalent models yielded identical results with the MIXED procedure in SPSS. Additionally, the GLMM analysis on error rates was conducted using the PROC GLIMMIX function in SAS.

The CADM (“Congruence among distance matrices”) (Legendre and Lapointe, 2004) and Mantel tests (Mantel, 1967; Mantel and Valand, 1970) were conducted on the similarity matrices using the routines CADM.global and CADM.post in package ape (Paradis et al., 2004) in R (R Core Team, 2013). One-tailed significance tests, corresponding to a positive association, were conducted for both the CADM and Mantel tests, following the procedure described in Legendre and Lapointe (2004). 99,999 permutations were conducted to assess significance for both tests.

### Conflict of interest statement

The authors declare that the research was conducted in the absence of any commercial or financial relationships that could be construed as a potential conflict of interest.
